# Thea: empowering the therapeutic alliance of children with ASD by multimedia interaction

**DOI:** 10.1007/s11042-021-11520-9

**Published:** 2021-10-30

**Authors:** Rita Francese, Michele Risi, Genoveffa Tortora, Francesco Di Salle

**Affiliations:** Department of Computer Science, University of Salerno, Fisciano, Italy

**Keywords:** Autism Spectrum Disorder, Empowerment, Chatbot, Multimedia Interaction, Therapeutic Alliance

## Abstract

The Therapeutic Alliance (TA) between patient and health provider (therapist or clinician) is one of the most relevant factors for the success of a therapy. In the case of people suffering from Autism Spectrum Disorder (ASD), the alliance is extended to all the people involved in their care (i.e., teachers, therapists, clinicians, relatives). In this paper, we propose a multimedia application named Thea for empowering the TA of children with ASD by improving the communication among the TA members, sharing guidelines, multimedia contents, and strategies to comply with challenging behaviors and progress with particular attention towards end-users who are occasional smart-users. A detailed process for empowering the TA members by enhancing the informed interaction among all of them is proposed and implemented. A vocal assistant also supports patients/caregivers and therapists in documenting their activity with the person with ASD by recording videos in a free-hand modality. After a contextual analysis based on Thematic Analysis Template, Thea has been implemented using a user-centered development approach. We performed three iterations involving the end-users. A user study is performed at the third iteration. Results of the user study revealed a positive attitude towards the application. In particular, the perception of empowerment of participants increased after the tool had been used. We also highlighted the guidelines and tools that may be adopted for empowering different kinds of patients. The first results seem to suggest that the use of Thea may increase the belief of the caregivers of a person with ASD to be able to better take care of her, in a more controlled and informed way.

## Introduction

Nowadays, the term *empowerment* is largely adopted in various professional fields, ranging from business, health, education, and psychology. It emerged during the ’70s, when Freire [15] stated that a person who has the right resources may critically perceive his personal and social situation, gain control and improve his life. This is particularly true in the health field. Many health policy programs favor patient empowerment [27] and encourage health care systems to be centered on the patient [32]. This goal is reached by adopting healthcare information systems designed to empower patients by positively influencing their behaviors that will act on their quality of life. Another factor that may improve the therapy results is the pursuit of the Therapeutic Alliance (TA). According to Horvath et al*.* [20], TA between patient and therapist is reached when they agree on the treatment goal and on the tasks to be performed to pursuit the goal, and reciprocal positive feelings are created.

The empowerment concept has been extended to all the family when it includes a child with a disability. This is particularly true for families with children with serious development disorders: family members have to be informed and participate in the planning and delivery of the therapy to their children [16]. The concept of TA too may be extended in this case to all the actors that take care of the child: family members, personal assistants, teachers, therapists, clinicians, and psychologists. The use of multimedia and mobile technology let us foretell future applications for moving therapy beyond the one-on-one encounter. A software system to empower the TA should first establish strict communication among all the alliance members for sharing the planning and monitoring the treatment results. This system should follow specific guidelines to be accessible to occasional smartphone users, who have different technological backgrounds and cultural levels [40].

The contribution of this paper is the following:

- propose a multimedia application, named Thea, for empowering the TA members (caregivers, patient’s family members and the patient, when possible) of a person with an Autism Spectrum Disorder (ASD) by sharing guidelines, multimedia contents and strategies to comply with challenging behaviors and progress.
- design the multimedia empowerment process addressed to the TA members of children with ASD;
- ease the Thea user interaction by offering a vocal assistant to support patients/caregivers and therapists in taking videos in a free-hand modality.
- assess whether the use of Thea empowers the Therapeutic Alliance perception;
- propose guidelines and tools for the analysis of an application for empowering the TA of other kinds of patients.

The paper is structured as follows. We discuss related work and background concerning empowerment, TA and multimedia interaction in Sect. 2; Sect. 3 introduces the empowerment goals of the TA; Sect. 4 describes the proposed approach concerning the empowerment of the TA of a child with ASD. Section 5 and Sect. 6 describe the definition of the empowerment goals and the prototyping cycle of the Thea development, respectively. Section 7 discusses the lessons learned. Finally, Sect. 8 concludes the paper and proposes future work.

## Background and related work

### Patient empowerment

In Europe, great relevance is given to patient empowerment through the European Patients Forum which promotes *“the development and implementation of policies, strategies and healthcare services that empower patients to be involved in the decision-making and management of their condition...”*. Empowerment is a multidimensional concept of which many definitions have been provided in the literature [5, 27]. Bravo et al*.* [5] designed a conceptual map of patient empowerment which has been derived through literature review and qualitative interviews with key stakeholders. The analysis identified that patient empowerment depends on two factors: behaviors and capacities which are strictly connected.

In the literature, many applications already exist supporting the patient in the auto-administration and self-monitoring of therapy [2]. In [23] an empowerment approach for people affected by diabetes is proposed. It is based on a self-management tool that enables the patient to specify his customized activities and his goals by considering medical recommendations. The usability and usefulness of that framework were also assessed.

Vitiello et al*.* [41] presented an empowerment methodology based on User Experience (UX) requirements. A contextual investigation is conducted to understand behaviors and capacities to be empowered and propose life quality improvements. A preliminary case study on elderly people has been discussed in [42].

### Therapeutic alliance & multimedia interaction

The relationship between the therapist and the patient has a serious impact on the results of the treatment [17]: it can determine treatment outcome, treatment dropout, and treatment attendance.

The alliance is composed of two factors: a *“personal alliance”* based on the interpersonal relationship created between patient and clinician, and a *“task-related alliance”* based on the sharing of goals, methods, focus, and *“depth”* of the treatment [21]. TA has a large impact on patient empowerment, as shown by Anderson et al. in [3]. The patient passes from being a passive recipient of care to become an active participant, establishing an equitable partnership with the clinician [18]. The patient provides to the alliance his knowledge on his status and his experience, while the clinician provides the knowledge on how to manage the patient’s status. The alliance lets the patient be more responsible for his care and participate in the decisions concerning his health through the development of self-esteem, confidence, and self-efficacy [28].

In the related scientific literature, several software applications have been proposed for enhancing TA between patient and clinician. As an example, in [25] patients with eating disorders adopt an app for self-monitoring and for patient-clinician linkage, which enables clinicians to access patient data anytime. Abrahamyan et al*.* [1] present a platform that supports the communication among people with disability and lets the clinicians monitor patient communication, store medical records for organizing group discussions and diagnosis.

Richards et al*.* [36] assessed whether the use of technology would enhance patient ratings of therapeutic engagement in the case of face-to-face therapy in psychotherapy. The adopted software platform, named goACT, includes *“appointment reminders, two-way pre-scheduled messaging (secure SMS, online, email), self-monitoring, secure journaling, online homework records, and online psychometric assessments”*. It emerged that the use of GoAct enhanced therapy.

Sun et al*.* [38] proposed a multimedia-based approach for helping caregivers and patients in the management of their self-care after a cancer intervention.

The parent-therapist alliance also highly impacts the therapy outcome for children with ASD. A family-centered intervention model is the key, with a strong emphasis on the collaboration between parents and therapists in the activities related to the planning and delivering of the therapy [10]. The de facto therapeutic role conducted by the educational institutions has been investigated in [11]. The TA between the therapist and the child is most effective when it supports the alliance of the network of relationships among therapists, parents, and teacher.

Map4speech [24] is a mobile app exploiting the Behavior Modeling Training (BMT) approach to teach the user to conduct a naturalistic intervention with children with ASD to enhance their functional communication. This application proposes predefined videos. In the app developed in our case study, all the contents are provided by the therapists and by the caregivers.

## Therapeutic alliance empowerment goals

The Therapeutic Alliance is present in all treatment approaches, with some discernible differences concerning how the alliance is conceptualized. Muran and Barber [30] noted that each one of the three most diffused treatment approaches (i.e., psychodynamic, cognitive-behavioral, and humanistic therapies) considered TA to be an important treatment variable and that the difference among approaches lies within which aspects of the alliance are emphasized and utilized in the therapeutic process.

In the case of people with ASD the therapeutic alliance is extended to all the caregivers, including teachers and therapists.

**Fig. 1:**
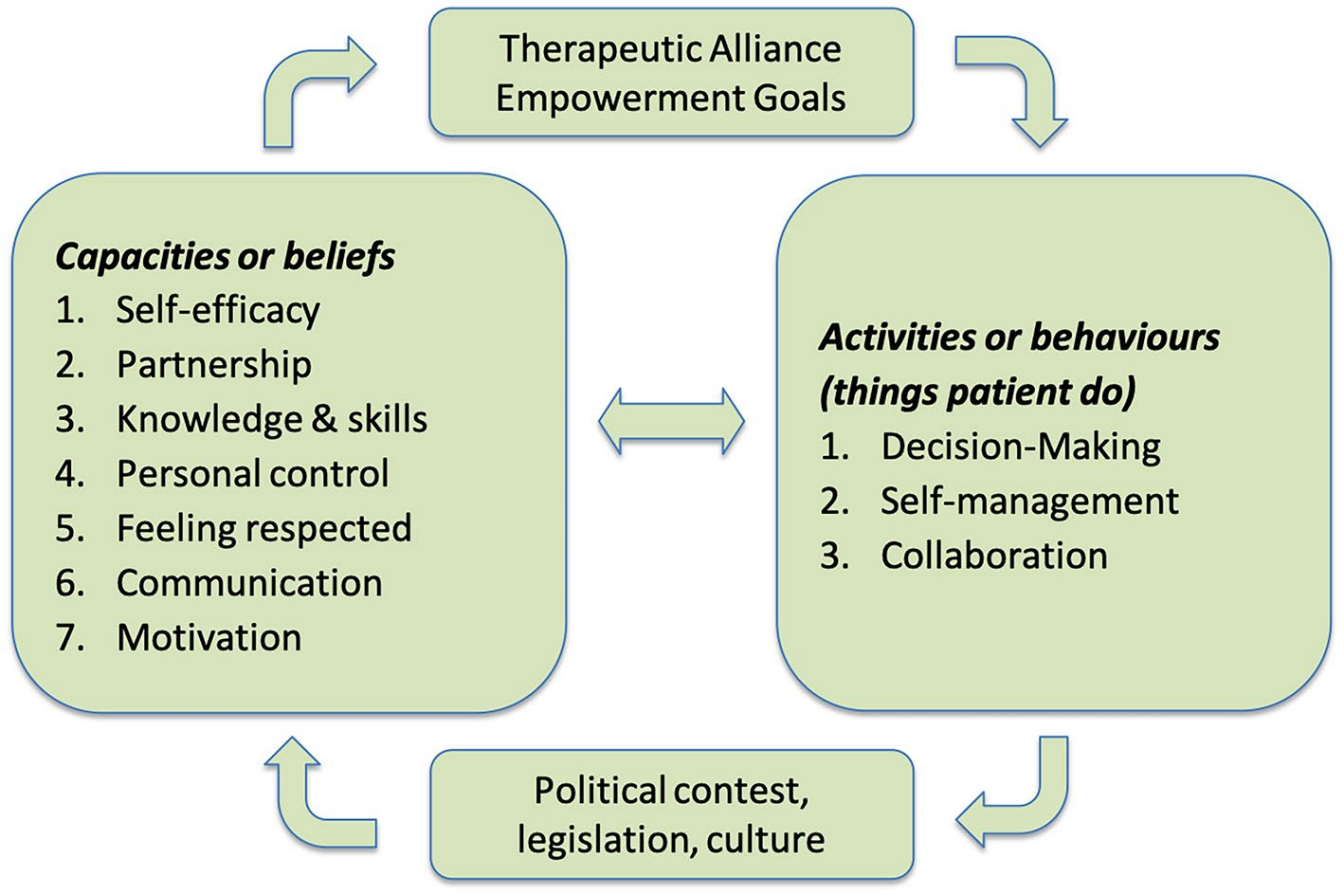
Therapeutic Alliance empowerment factors

Their empowerment may be reached by acting on several factors involving behaviors and capacities [5]. Figure 1 shows the capacities and behaviors which promote TA empowerment and the relationships with environmental influences. We derive them by [5] and [22]. The description of the various dimensions is provided in Table 1.

**Table 1:** Therapeutic Alliance empowerment goals

Capacities or beliefs	
Self-efficacy	Self-efficacy refers to individuals’ assessments of their effectiveness or competency to perform a specific behavior successfully [34]. *It is concerned not with the skills one has but with judgments of what one can do with whatever skills one possesses* [4].
Partnership	Adopting a partnership style within the healthcare relationship lets the patients be the experts by experience, while clinicians and therapists are the experts in the knowledge of the disease.
Knowledge & Skills	Enabling factors to be able to engage with the healthcare provider.
Personal control	Based on the person’s belief that the patient has the power to produce positive results.
Feeling respected	When a patient (or her caregivers) feels to be respected by the health providers communication and motivation improve. When patients (or her caregivers) are respected and they trust providers, they more likely seek care, establish a more strict relationship with the provider and follow the therapy.
Communication	The communication among the members of TA is good. They share their experience in patient support/advocacy group for sharing common goals, also through social network.
Motivation	Motivated patients increase their self-efficacy and obtain better results.
**Activities or behaviors**	
Decision-making	Actively make informed decisions about their own health and choose realistic objectives.
Self-management	Patients have in charge their self-management behavior and adhere to a prescribed therapeutic regimen.
Collaboration	The patient works together with the TA members in pursuing mutually negotiated goals.

## The proposed approach

Children and adolescents with neurodevelopmental disturbs may be shy, aggressive or obsessive, and have school difficulties. Many different psychopathologies are associated with these problems and all require personalized rehabilitative and educational interventions. Every child is a specific case. Only concerning the problems related to autism in the United States 1:68 child belong to the spectrum. In Italy 20% of children have learning disturbs of various origin. It becomes essential to properly take care of patients with appropriate rehabilitative interventions as soon as possible. Day-by-day rehabilitative activities must be defined. The most adopted interventions are based on the Applied Behavior Analysis (ABA)^1^ therapy.

ABA therapists and experts recognize that the therapeutic relationship provides a significant contribution to patient improvement. Collaboration and teamwork are among the most relevant empowering factor [6]: therapist and client (patient or his caregivers) work together, as a team, to identify the client’s problems as well as to identify solutions. The treatment plan should be explored together, by experimenting with strategies and evaluating the results, and reformulating the plan. All the people that take care of the children have to actively take part in the planning.

The goal of this paper is to propose and assess a multimedia application, named Thea, for empowering the TA members (e.g., caregivers and patient’s relatives) of a child with ASD by sharing guidelines, multimedia contents and strategies to comply with challenging behaviors and progress.

**Fig. 2:**
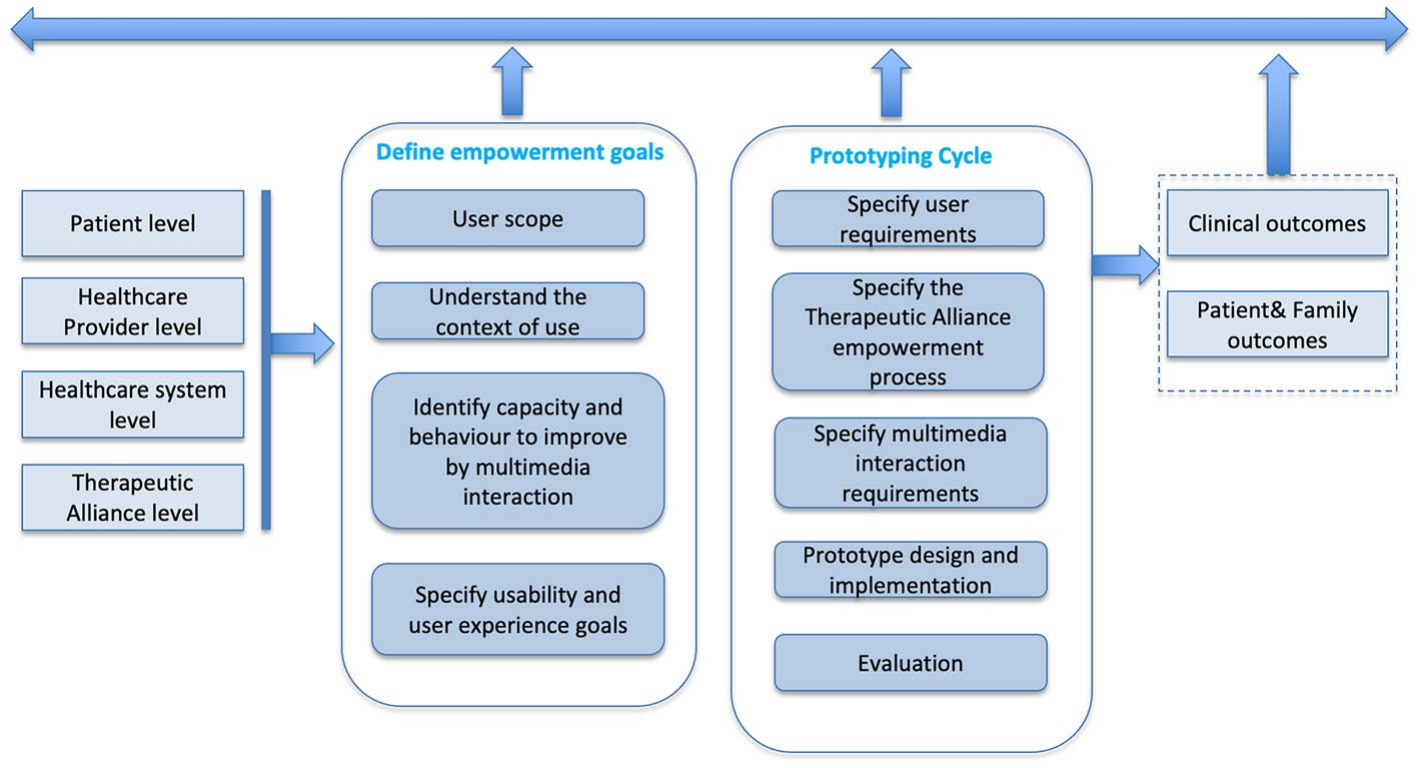
The adopted user-centered methodology

User-centered design is largely adopted in e-health application development to ensure effective outcomes [37], and is counseled by the World Health Organization (WHO). We adopted the methodology shown in Fig. 2 that introduced in a traditional user-centered design methodology aspects related to the TA empowerment. In the following, we detail how the activities of the methodology have been addressed in the considered case study. In particular, the Prototyping cycle has been conducted three times.

The following two sections describe the definition of the empowerment goals and the prototyping cycle for the development of Thea.

## Define the empowerment goals

The first step to perform is the understanding of the empowerment levels of the Therapeutic Alliance circle, including the provider(s) of the health service. As depicted in Fig. 2, this step is composed by several activities, described in the following.

### User scope identification

First, we identified the target users. They are Italian people members of a circle which cooperates in the care of a patient with ASD. Clinicians and therapists are the professionals involved, while caregivers are parents, teachers, personal assistants, and the patient when autonomous enough. The target users are of different technological backgrounds and social environments.

### Understand the context of use

The understanding of what the domain of the community to be empowered is may be performed by querying the interested people on which the process of managing their care is and how it is conducted. This phase is composed of two steps:

1. Interviews for caregivers and professionals, where we selected some users representative of each TA user category, proposed a demographic questionnaire concerning gender, cultural level, employment, and technological level and conduct a semi-structured interview useful to get feedback and write a survey;
2. survey, dispensed to several users of each category to understand how the aspects that the interviewed users highlighted are actually managed, what is wrong and what is positive, and which their empowerment level is.

#### Interviews for caregivers and professionals

*Method.* We decided to divide the members of the TA of a child with ASD into two groups of users: caregivers (family members, teachers, personal assistants) and professionals (therapists, clinicians, psychologists) and designed an interview for each group. We adopted Thematic Analysis Template (TAT) for structuring the interviews, a qualitative method to identify patterns, themes, and interpretations in data [29]. TAT uses template documents, which list a set of initial themes that have to be discussed.

*Study material.* We designed two templates: the template in Table 2 for the caregivers and the one in Table 3 for professionals. The templates were manually filled in by all the researchers, who all agreed on the selected themes and also registered the discussion.

*Interview to caregivers.* We interviewed two mothers (M1 and M2), two teachers (T1 and T2), and a personal assistant (PA). The interview was conducted by following the template in Table 2. The following findings were collected. The ids of the questions we proposed in the survey described in Sect. 5.2.2 are associated in bold to each relevant sentence.

**Table 2:** Thematic Analysis Template for caregivers (family members, teachers, personal assistants and the patient)

**ID**	**Theme**	**Discussion**
1	Shared decision-making and Integration of patients’ views	a. Describe how the therapy is managed and delivered by your provider.
		b. Reveal how the provider involves you in the decision-making process.
		c. How are the goals of your therapy fixed?
		d. Describe how your knowledge on your disease is helpful in the communication with your provider.
		e. Reveal if you feel respected during the communication with your provider.
		f. Describe the pros and cons of the collaboration and how it may be improved to reach a successful Therapeutic Alliance. Which support may be provided by the technology?
2	Support to self-care and self-management	a. Please, describe how you adhere and manage by yourself a prescribed therapeutic regimen and the activities of self-management and self-care you perform.
		b. Reveal what the positive and negative aspects were occurring when you/the patient try to solve his health problems when they happen.
		c. Describe your perception on your (the patient) capacities of producing positive results and make informed decision about your health.
		d. What were you/they not able to do in your self-care and self-management? What helped you/them to get what you/they need? Does the provider help you to be autonomous in your self-care and self-management?

**Table 3:** Thematic Analysis Template for clinicians, health managers and health researchers

**ID**	**Theme**	**Discussion**
1	Communication	What do you think people need to communicate/be informed on the therapy care service?
2	experience	Reveal what the positive and negative points were encountered when the various actors (physician, therapist, teacher, caregiver, patient) cooperated in the therapy subministration.
3	Improvements	Reveal what can be done better to improve the previous negative points
4	Empowerments	Describe health interventions or health services approaches that promote alliance empowerment. Which support may be provided by the technology?
5	Report	What patient reported outcomes do you think are useful to assess the therapy results?

*Shared decision-making and integration of patients’ views.*

- The general procedure of take care and subministrate the therapy is the following: the ABA expert performs a first meeting with the parents and evaluates the child through standard tools. Successively, the expert defines an operating relationship which is shared and discussed with the therapists, the school, and the family. Therapists then work with the patient for the weekly established number of hours. The treatment is monthly monitored, through video and data.
- When the procedure is correctly actuated, parents share the goals with the ABA expert as follows: after having evaluated the child the expert asks the parents what are the things the daughter prefers to do. Teachers generally are involved in the meeting via Skype. PA is informed by the child’s mother on the activity to do and informally by the therapist when he meets him.
- The initial goals should be established by involving all the caregivers. The goals are divided into the various cognitive areas, communication, social skills, and academics, autonomy development, such as fine motor dexterity, domestic capabilities, grooming, hygiene, and competence at work.
- All the interviewed agreed that the background is fundamental for the self-management of their children and also for discussing with their providers.
- The daily communication among the circle members generally occurs via Whatsapp (**Q6**). Caregivers have to take a diary where they annotate the progress, the reached level of autonomy, and the challenging behaviors (**Q10**). Rarely therapists produce videos of the therapy. M1 would like something to explain to the other family members how to interact with her daughter. T1 would like to be updated more frequently on the progress and the problems of the child (**Q5**). M2 says that when a therapist or a teacher is substituted, the new entry is not informed on the child’s problems and does not know how to manage her daughter, therefore producing negative effects.

*Support to self-care and self-management.*

- Once the patient level has been assessed, the goals are identified and for each goal, a list of tasks is defined. Each task is then analyzed and subdivided into its components. After having learned a competence in the controlled setting of the therapist, ABA is extended to the real settings, such as home, school, and external environments. So the caregiver follows the guideline by successively executing each step of each task.
- One of the mothers follows the videos she has taken while the therapist manages her daughter and spreads them to the teacher and her Personal Assistant (**Q9**). To try to understand what causes a challenging behavior (e.g., self-injury) and how to prevent it, parents have to collect information related to the problem behavior and understand antecedents (what happens before a behavior occurs) and consequences (what happens after the behavior). Parents have to complete the ABC chart every time a problem behavior occurs (**Q11**). See [14], where an example of the ABC Chart is shown. The challenging behavior occurring has to be communicated to the other people of the circle (**Q5**). To this aim they generally use Whatsapp.
- People who are better formed are more aware and able to make an informed decision and to have a positive influence on the child’s results since they always actively participate in the therapy planning. Generally, the participants perceived that their opinion is taken into account.
- Participants would like to have more precise guidelines and more activities to perform (**Q9**); more contact with the provider (**Q12**). The use of Whatsapp for communicating merges different kind of information, including greetings, and make difficult to search the data.

*Interview to medical staff.* The interview has been conducted by following the template in Table 3. We involved a neurologist, a psychologist, and a therapist of private centers. Results are summarized in Table 4.

**Table 4:** Results of the TAT analysis for medical staff

**ID**	**Theme**	**Discussion**
1	Communication	All the participants agreed that the involvement of all the caregivers of a child with ASD is one of the most important success factors, because improvements may be reached if the therapy is applied every day, all the day. They periodically meet the caregivers (generally monthly), including teachers. The clinician has many patients and answers only in case of real difficulty (**Q12**).
2	experience	Clinicians lamented that often the caregivers forget to annotate relevant information, such as the one concerning challenging problems (**Q11**). Many times the teachers do not have the right preparation to manage difficult cases with challenging behaviors. Often parents do not accept the disturbance of the child, often for cultural problems, so they do not participate in the appropriate way (**Q12**).
3	Improvements	All agree on the need of having a medical record in common with all involved people, in such a way to share signs of progress (**Q7**,**Q8**). The therapist would like to have also an electronic diary with videos (**Q10**). The psychologist agreed, but he also would like to have an immediate report on the challenging behaviors (**Q5**). Concerning the production of audio-video guidelines, for the therapist is difficult to make the therapy and, at the same time, to take videos.
4	Empowerment	The caregivers may be empowered if they are more informed. In any case. they should share the goals with the providers and also learn by-examples how to conduct self-management activities at home while respecting the child’s attitudes (**Q9**).
5	Report	All the participants agreed that the task activity should be measurable. The most relevant metrics are the autonomy level on which a child may perform a task (e.g., with total physic aid, with partial physic aid, with vocal aid, autonomously). Two other relevant measures are the time for accomplishing the task and the task frequency (**Q14**).

**Table 5:** Questionnaire for the Therapeutic Alliance actors of a children with ASD

**ID**	**Question**
Q1	I use the smartphone for communicating with the other people involved in the care of the special needs person.
Q2	I use emails for communicating with the other people involved in the care of the special needs person.
Q3	I use apps (e.g., Whatsapp, Messenger, Instagram, Facebook) for communicating with the other people involved in the care of the special needs person.
Q4	I use Face-to-face for communicating with the other people involved in the care of the special needs person.
Q5	It is useful to promptly communicate with the other people involved in the care of the special needs person.
Q6	It is useful to use a single means of communication.
Q7	It is useful to have the patient’s information needed for a diagnosis all in a single place.
Q8	It is useful to have the material to evaluate the patient progress in a single place.
Q9	It is useful to have at disposal examples of the activity conducted during the therapy for all the caregivers.
Q10	It is useful to have an electronic diary where to annotate the challenging behaviors.
Q11	It is useful to collect data to compile the ABC table *“antecedent- challenging behavior - consequent”*.
Q12	I’m satisfied of the communication between the people that take care of the special needs person.
Q13	If no, describe these problems (open question).
Q14	(only for clinicians and therapists) Which task progress metrics do you consider relevant? (suggested: Autonomy Level, Frequency, Time to accomplish a task, more than one answer is allowed and additional may be added).

#### Survey

*Method.* The interviews conducted by using the TAT analysis are transcribed and the most relevant aspects are identified. Then, a question is associated with each relevant theme, identified by the question id in bold in Sect. 5.2.1.

*Study material.* We prepared the questionnaire shown in Table 5. The answers were scored with a Likert scale from 1 (not at all) to 5 (very much). We also collected demographic information of participants. Questions Q1-Q4 are devoted to the understanding of which communication means the participants usually adopt for communicating with the alliance members. The other questions are derived from the TAT analysis to further verify their real needs.

*Study context.* Participants were 101 end-users belonging to associations of family members with an ASD child, 65.5% woman, the other men. Their roles in the patient alliance were: 31% family members, 27.6% psychologists, 13,8% therapists, 10.3% teachers of children with special needs, 6.9% personal assistants, and 3.4% clinicians. 65.5% were graduated. All the users had a smartphone and used Internet. 31% made large use of social networks, 17% made use of e-Commerce for buying products, and 27% read online news. Only 13% played online games. 44.8% were accustomed to installing and using new apps on their own smartphone. So we can conclude that most of them moderately used a smartphone.

**Fig. 3:**
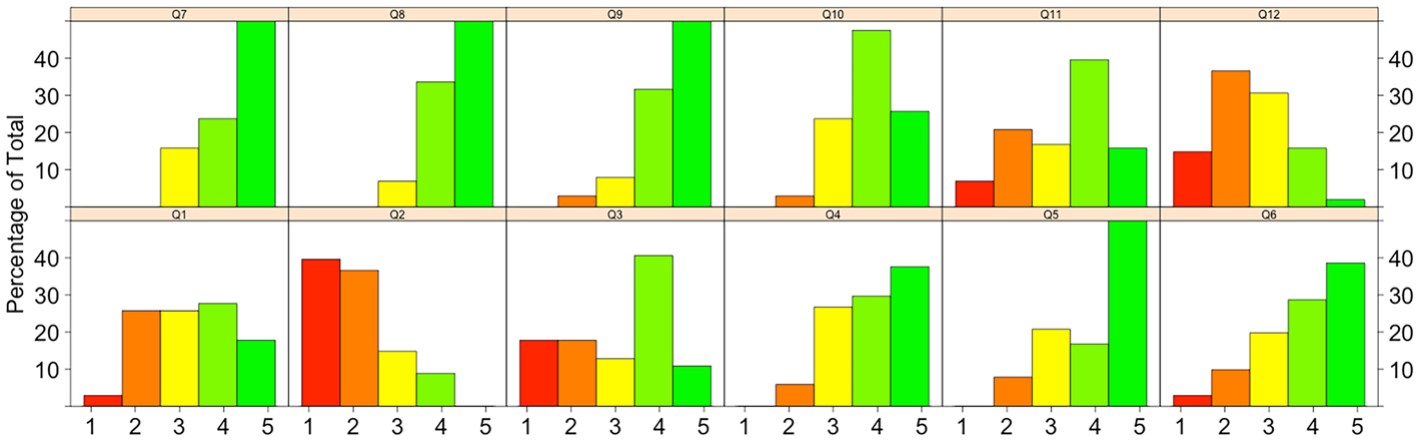
The survey results

*Results.* The histograms in Fig. 3 summarize the answers provided to the questionnaire in Table 3. Concerning the means participants use for communicating with the other members of the circle, 46% uses the phone (**Q1**), email only by 9% (**Q2**), 51% uses Whatsapp (**Q3**) and 68% communicate face-to-face (**Q4**). 72% consider useful to have the possibility of immediate communication with the circle members (**Q5**), while 67% judges useful to employ only one of the communication means (**Q6**). Also to collect all the material in a single place for diagnosis purpose (and for evaluate the patient progress) is considered useful by 84% of participants (**Q7**) (93% **Q8**). Most participants (89%) were favorable to the idea of having examples of the therapy subministration available for all the circle (**Q9**). 73% of participants agreed on the need of having an electronic diary at disposal where annotating the challenging behaviors (**Q10**), while the 55% of them were interested in filling the ABA information (**Q11**). Only 18% was satisfied by the communication among the people involved in the patient care (**Q12**). The open question (**Q13**) collects their concerns: some clinicians and therapists scarcely communicate the therapy objectives and the way to reach them and the caregivers have a sense of loneliness. Some of them do not take part in the therapy planning. They signal the lack of group sessions with all the involved parts and the lack of information of the clinicians on the patient status. On the other side, therapists and clinicians lamented having a very high workload with a lot of patients and that often patients do not follow indications and do not have the skills for providing a useful contribution. Concerning the task performance metrics (**Q14**), all the clinicians agreed that the three suggested metrics are essential.

### Identify capacity and behavior to empower with multimedia interactions

Table 6 shows the relevant tasks we identified from TAT interviews and the questionnaires. The table also shows for each task the behaviors that are modified by the process and the capability which should be improved. We also identified which activities need the support of multimedia features.

**Table 6:** Task Analysis and related empowerment objectives

**Task**	**Description**	**Decision Making**	**Self Management**	**Collaboration**	**Multimedia**
T1	Adherence to the treatment objectives	K&S	SE, PC		No
T2	Learning how to perform the therapy	K&S	SE, PC	MOT	Yes
T3	Reporting task execution		PC	COM	Yes
T4	Reporting challenging behavior			COM	Yes
T5	Monitoring progress	PC	K&S		No
T6	Communicating with the other alliance members	K&S		MOT	No

The analysis of the contextual inquiry revealed that T1 is a task that has to be mainly conducted in F2F modality, or in video-conference, while the other tasks may be supported by multimedia technologies. The therapy of each child is customized in terms of tasks and measured in terms of autonomy level, frequency, and accomplishing time of each task. So pre-defined material may be not appropriate for each circumstance. The idea of providing video/audio guidelines created ad-hoc by the therapist appears to be the most appropriate. Similar functionalities may be adopted at home or at school.

### Specify u sability and user experience goals

Before starting development, Usability and User Experience goals have to be defined by taking into account that people involved in the TA may be occasional smartphone users and with different cultural backgrounds. Thus, this kind of application has to be particularly easy to use to avoid the risk of abandonment. Also, clinicians and therapists have to find it appropriate to use during clinical practice. So, also for them, there is the need to immediately find the required functionality to save time. We identified the following usability goals for all the actors: *(1)* Effectiveness, the application has to provide a guide for the Thea members and help them in the therapy activities; *(2)* Efficiency, the application has to help the caregivers in performing the patient tasks efficiently; *(3)* Satisfaction, the application should be judged easy to use and helpful by every Thea user of whatever background. We also are interested in evaluating the global User Experience as relevant evaluation criteria. These goals have to be pursued especially for occasional users in the use of smartphone since the technological background of many TA members is low.

We decided to adopt for Thea a minimalist interface style for easing the interaction. Indeed, the app should be designed for limited and split attention [39], because when the circle members are involved in activities with the child with ASD they should be able to multitask. Cognitive aids should also be particularly considered, e.g. to remind a user of what she has to do because the attention of the users may be limited or they may be stressed. Concerning the interface colors, we decided to follow the guidelines traced in [8] that recommend exposing end-users to blue color which positively impacts on psychological effects and social interaction. We understood that it is very relevant to minimize the number of interaction points, especially when recording challenging problems (caregivers) or during the therapy recording (therapists, caregivers) where the end-user attention is addressed to the child. User registration should also be minimal, we do not expect family members having a lot of problems to spend a lot of time in data entering. The use of a user-centered design approach should enable us to get better human-computer interaction.

## Prototyping cycle

In this section, we describe three prototyping iterations. The first is centered on the design, the others mainly on development activities.

### First Iteration

*Specify user requirements.* We conducted a focus group with the same users involved in the interview, who were informed that all the discussions related to the study were confidential. The objective of this session was to identify the list of requirements to empower all the TA members in such a way to adopt the same procedure for managing the patient and conducting her rehabilitation activities with a uniform approach. We started from the empowerment goals summarized in Table 6. This session produced as a result the list of requirements reported in Table 7.

**Table 7:** First iteration requirements

**Actor**	**Description**
Clinician	manage the diagnosis data, the objectives and describe the challenging problem of a patient.
Therapist	manage the tasks to be performed;provide guidelines on how to perform each task.
Therapist, caregiver	collect reports on the performed activities;provide reports on challenging behaviors.
Circle	examine the patient record;monitor signs of progress; communicate on a Therapeutic Alliance common chat; receive notifications when events happens; participate in a community of users for sharing experiences.

One of the objects of discussion was related to the privacy of the contents: participants all agreed that they would like their data to remain visible only to the circle. When we insisted by explaining that information may be useful for statistical purposes or for helping others in case of success stories some of them said to be available to share their data only with medical staff. So we decided that a user may decide to choose among three different types of visibility, as reported in Table 8.

**Table 8:** Visibility types

**Actor**	**Description**
All	All the people accessing Thea may see the user data.
Health providers	The data will be available to all the providers registered in the system for further analysis.
Circle	Data are visible only to the members of the Circle.

*Specify the Therapeutic Alliance Empowerment Process.* By considering the empowerment goals, the inefficiencies, and the identified requirements, during the focus group we also designed the patient management process related to TA of children with ASD, in such a way to empower the participation of all the members of the alliance.

*Method.* We adopted a scenario-based description supported by a model of the process represented by an activity diagram with object-flows and swimlanes, where the $<<Multimedia>>$ stereotype is adopted for denoting an activity which requires a more specific multimedia interaction design.

**Fig. 4:**
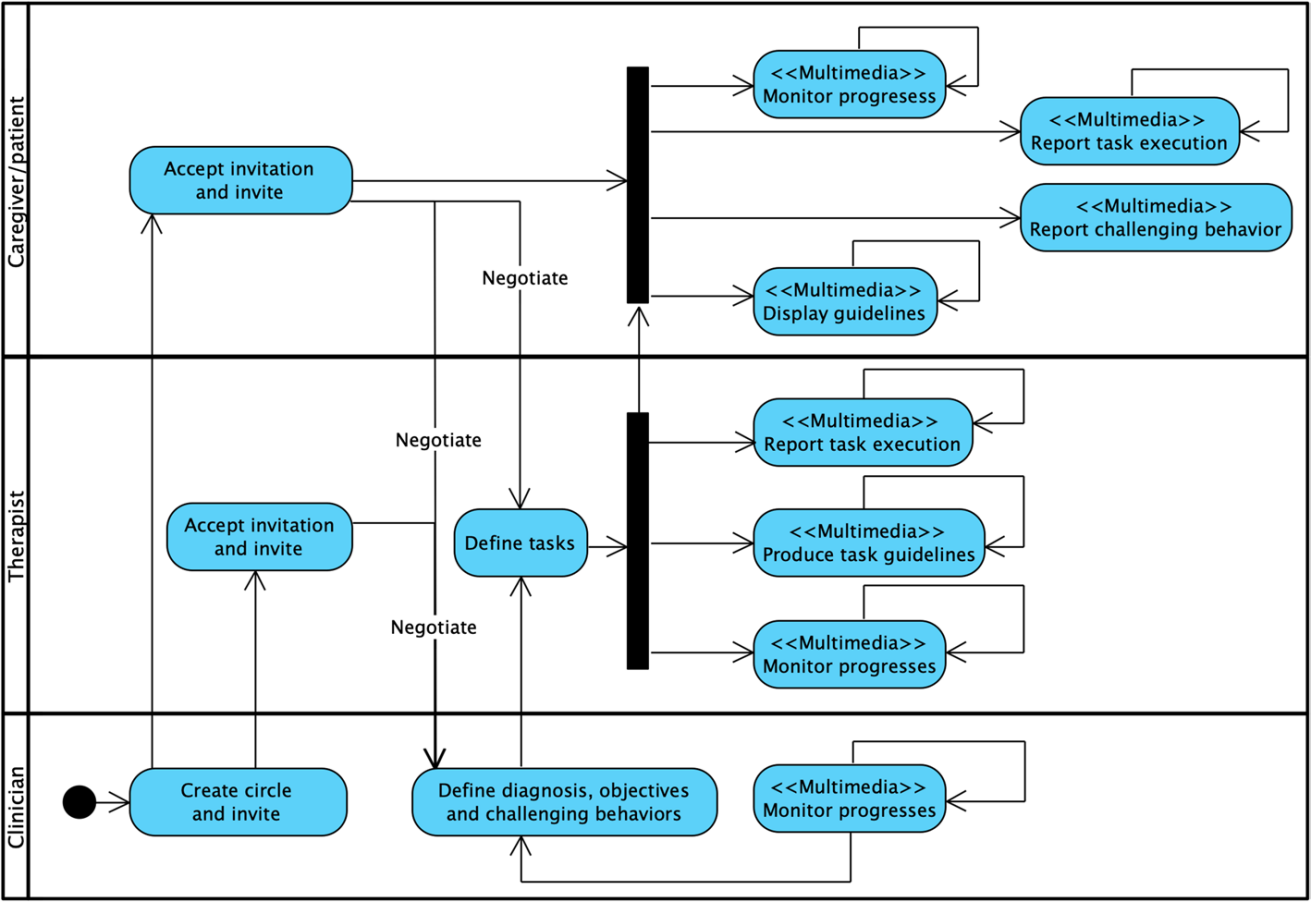
Therapeutic Alliance empowerment-driven process

**Fig. 5:**
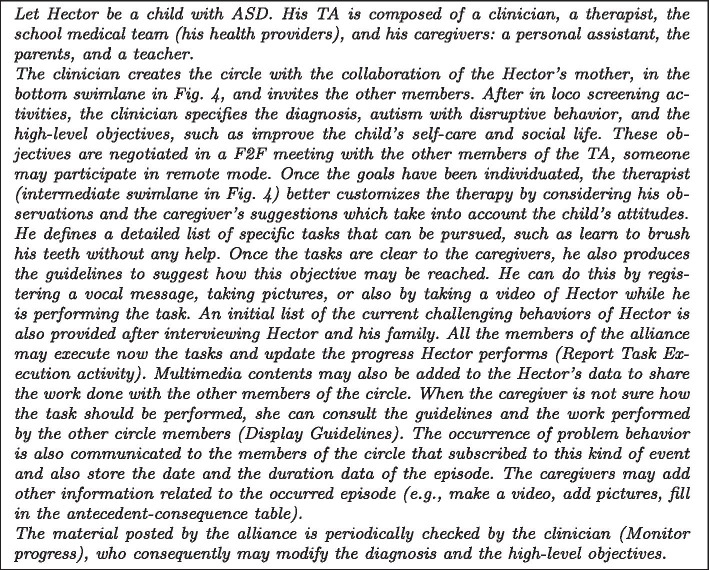
Scenario of the Therapeutic Alliance empowerment-driven process

*Process design.* We provided a scenario-based description of the process (reported in Fig. 5) and the activity diagram in Fig. 4 representing the articulation of the process in activities. We adopted a repository-based approach: all the information of a patient (the patient record) flows in a unique repository which is shared among all the TA members.

**Fig. 6:**
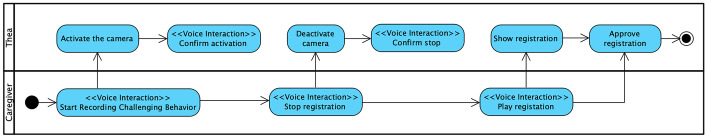
Model of the Challenging Behavior Reporting process

*Specify multimedia interaction requirements.* *Method.* We compiled a form for each multimedia task identified in Fig. 4. The task is modeled by an activity diagram with object-flows and swimlanes. Table 9 provides an example of task specification and the associated activity diagram is shown in Fig. 6.

*Interaction modality.* We decided together with the end-users to use both the traditional touch-based interaction and a female chatbot named Thea for providing support to the member of the circle when managing multimedia content during therapy. A female-gender chatbot has been chosen because women are considered friendlier [19].

**Table 9:** An example of multimedia task description

**Item**	**Description**
Task	Report challenging behavior activity.
Actor.	Patient/caregiver/therapist.
Goal.	Report challenging behavior.
Control	The user controls the challenging behavior recording by interacting with a vocal chatbot. Also the mobile device GUI supports this operation.
Context	Patient’s home or in an external environment.
Modeling	The activity diagram with object flow describing the multimedia interaction of the Report Challenging behavior is reported in Fig. 6. The actors are the caregiver and the chatbot Thea which may display multimedia content on the user’s smartphone or a TV.
Tool	A Google Home communication point may be positioned in each room together with a webcam. The interaction may also occur through a Google assistant on a mobile device.
Feedback	Caregivers appreciate this solution a lot for collecting data on challenging behaviors which allows them to maintain their hands free for intervening.

**Fig. 7:**
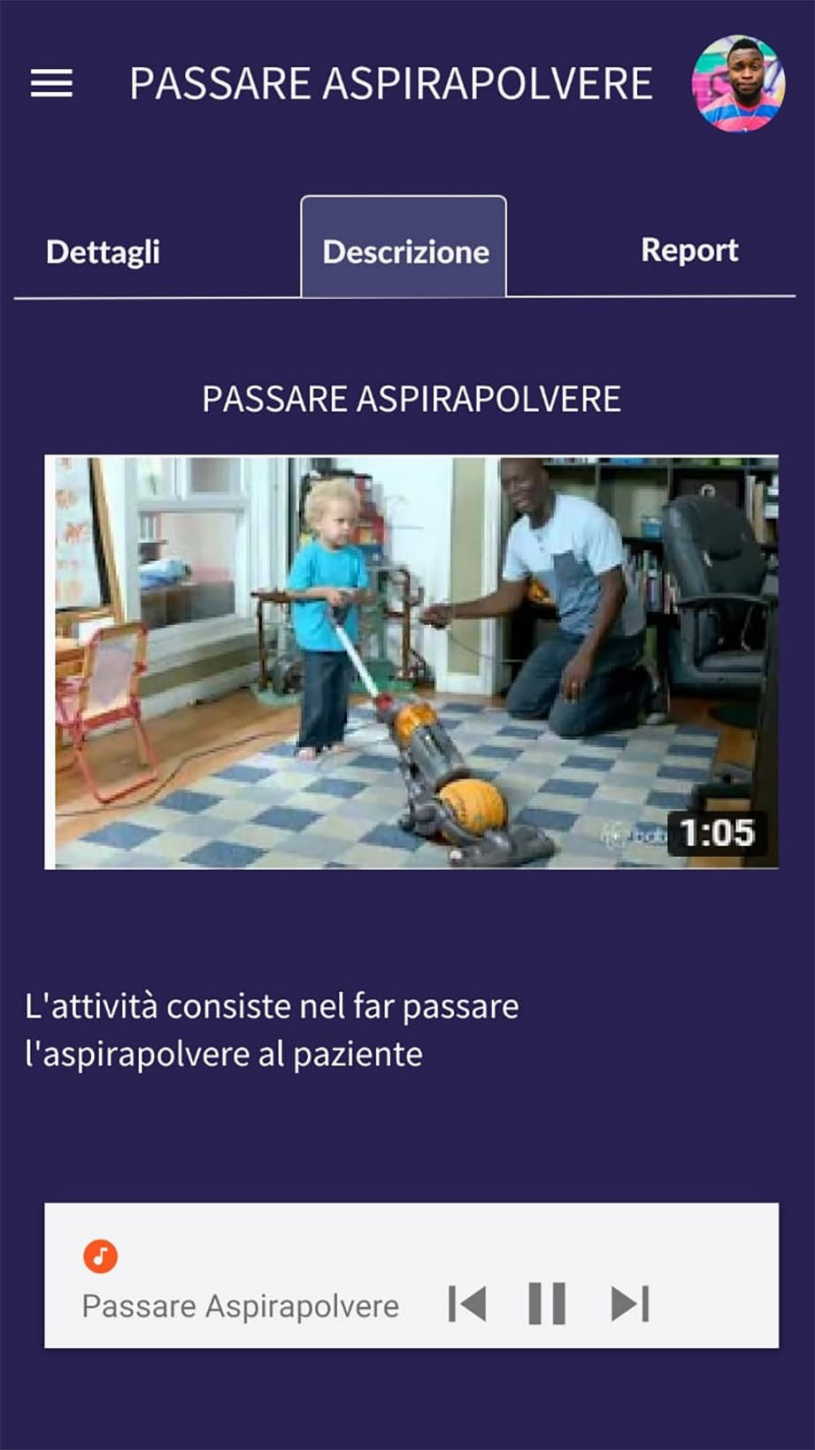
First prototype screens

*Prototyping.* During the focus group, we designed an initial prototype to better support the requirement collection. It has been shown to the participants when all the suggestions were collected, for not to influence them. Some of the final screens resulting from the session are shown in Fig. 7. Screens are in Italian because they are devoted to Italian people. The scale for measuring progress was also better defined (autonomy grade, time to accomplish the task, and frequency). Participants did not appreciate the dark background and asked to see a white version.

### Second iteration

The second prototype has been developed taking into consideration the suggestions collected during the previous interaction. Details are summarized in Table 10.

**Table 10:** Second iteration report

**Activity**	**Description**
Prototyping	A running prototype has been developed using the Angular-Ionic-Cordova technology for the development of the mobile application. Ruby on Rails and PostgreSQL are the adopted server-side and DBMS technologies. The choice of using multi-platform technology was due to the need of having the application available in both Android and iOS in a short time.
Evaluation	A heuristic evaluation was conducted with a group of 4 HCI experts of the University of Salerno, two of them were parents of a child with ASD, and a therapist. The therapist asked to be able to document challenging problems also by audio. Concerning usability, some buttons were not homogeneous. Some participants manifested difficulty in understanding without help in the use of the application. The colors depicting the levels of a challenging problem were too marked. Some functionalities were difficult to find and this increased the cognitive load.

### Third iteration

The third iteration is mainly concentrated on the improvement of the user interaction, as reported in Table 11. The prototype evaluation is deeply described in the next section.

**Table 11:** Third iteration report

**Activity**	**Description**
Prototyping	To assist caregivers in the use of the app, we introduced the chatbot not only for the management of multimedia content during therapy but also as an app assistant, i.e., to explain the app functionalities and help the user in finding the needed features. The chatbot clearly says which tasks it can perform. These tasks should be simple and not ambiguous. We enable people to both interact by the GUI and the vocal input. The chatbot starts by pressing a button always available on each screen of the application or by saying *“Hey, Thea”*. When the user dictates a command, the chatbot chat is opened and the dialog is traced (for example see Fig. 8c). Automatic message reply is done by a rule-based chatbot based on Dialogflow.
Evaluation	A lab-based user study has been conducted (see Sect. 6.4).

https://dialogflow.com

**Fig. 8:**
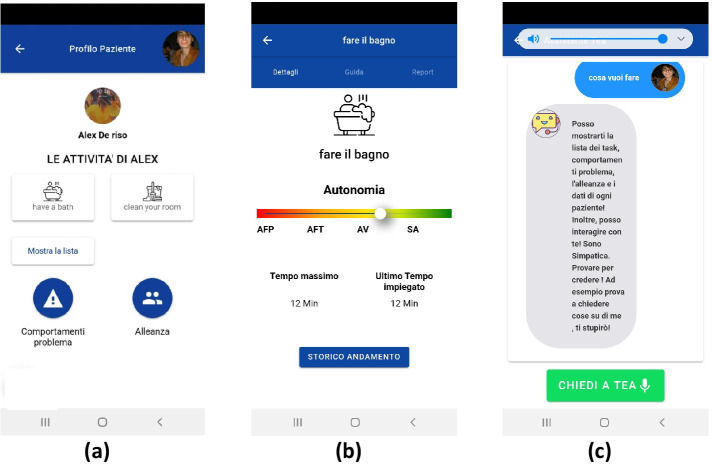
Second prototype iteration (**a**) and (**b**) and the chatbot added in the third iteration (**c**)

### Third prototype user study

We conducted a lab-based user study for the third Thea prototype in which we investigated the following main Research Question (RQ1):

*RQ1: does the use of Thea empower the Therapeutic Alliance perception of caregivers?*

To assess this question we investigated if there exists an effect (either positive or negative) of the use of Thea on the perceived Capacity-Belief of the participants. We also aim at assessing the Usability and the User Experience of participants globally and concerning their capability of using smartphones. So we investigated also the following research questions:

*RQ2: does experience in using a smartphone impact the usability of Thea?*

*RQ3: does experience in using a smartphone impact the User Experience of Thea?*

#### Experimental units

Participants were 31 caregivers who voluntarily took part to the study. They were all caregivers of a child with ASD. They were recruited through associations of families with an autistic child. We collected demographic data and information on participant’s technological skills through a pre-questionnaire. The average age of the caregivers was 45 (11.3 standard deviation). The pre-questionnaire revealed that 18 were skilled smart users (they frequently used the smartphone for sending emails, taking videos, surfing on Internet, e-Commerce, watching TV and listening to the radio, making video calls, installing new apps), the others were occasional smartphone users (they mainly used the smartphone with a chatting app and for making telephone calls, rarely used the device for surfing). 13 of them had some college education, one had a Ph.D., the others had a Bachelor’s degree.

#### Experimental material and tasks

As an experimental object, we used the third version of the Thea prototype. It has been pre-charged by the data of a circle composed of four caregivers, a clinician, and a therapist. Tasks related to personal care have been added with the associated guidelines.

To collect the user capacity-belief perceptions we used the Questionnaire in Table 12, for the assessment of the Empowerment perception, derived by [22]. Answers are rated on a 9 point Likert Scale from 1 to 9 (strongly disagree to strongly agree). The reliability of the modified questionnaire is assessed in Appendix A.

**Table 12:** Capacity-Beliefs Empowerment Questionnaire

**ID**	**Question**
S1	I am sure to be able to solve my therapeutic tasks (Self-Efficacy).
S2	I have an active partnership with my Therapeutic Alliance group (Partnership).
S3	I have enough information for managing my therapy (Knowledge & Skills).
S4	I am sure to be able to solve my therapeutic tasks (Personal Control).
S5	I feel to be accepted and well supported (Feeling Respected).
S6	I am satisfied of the communication with my Therapeutic Alliance group (Communication).
S7	I feel to be involved in my health care (Motivation).

The lab was equipped with a Philips Smart TV 7304 and a Reolink Argus 2 webcam. Thea was running on a Samsung S10e Android mobile device.

As experimental tasks, the caregivers had to perform the tasks in Table 6. They received a sheet on which the description of the tasks were reported. An example of task description is reported in Table 13.

**Table 13:** Example of task set for T2 - Reporting Task Execution

**Task**	**Description**
T4	Imagine you are in your dining room with your child. Your child is manifesting a challenging behavior. Ask Thea to register the video through the webcam, consult the registration on TV and approve it for saving.

After each task, to measure task-performance satisfaction, the sheet proposed the following question, which is scored on a 7-step Likert scale ranging from “very difficult” to “very easy”.

*PT. How difficult or easy did you find this task?*

For collecting the User Experience perception we selected the UEQ questionnaire [35], composed of 26 items grouped into six scales: Attractiveness (AT), Perspicuity (PER), Efficiency (EFF), Dependability (DEP), Stimulation (ST), and Novelty (NOV). Attractiveness concerns the aesthetics of the application, Perspicuity represents the easiness of understanding the application, while dependability concerns how the app seems trustworthy. Stimulation represents the joy of using the tool and novelty describes the perception of how innovative is the product. Each item has assigned two terms, such as fast/slow. Items may be scored by using a 7 point Likert scale, ranging from -3 (fully agree with the negative term) to +3 (fully agree with the positive term). The original User Experience Questionnaire^2^ was in German, but there are available translations in 20 other languages, also in Italian. We adopted the Italian version. We also add an open question for free-handing comments.

#### Hypotheses and variables

Concerning user capacity-belief perceptions we considered an independent variable named Order, a categorical variable with two values: PRE and POST collected by subministrating the same questionnaire in Table 12 before and after the experiment.

A dependent variable is considered for each capacity-belief: Self-Efficacy (SE), Partnership (PAR), Knowledge & Skills (KS), Personal Control (PC), Feeling Respected (FR), Communication (COM), Motivation (MOT).

- H0$_{X}$: There is no statistically significant difference between the PRE experiment and POST experiment to X.

where X is one of the considered dependent variables. Because we could not postulate an effect of the use of Thea in a specific direction on the perceived empowerment of TA, either positive or negative, our alternative hypotheses are two-tailed.

Concerning the measures of Usability, we considered as an independent variable the use of smartphone experience, a categorical variable with two values Not Smart User (NSU), Smart User (SU). We follow the approach proposed by Ferreira et al*.* [12] and considered as dependent variables:

- *Effectiveness*, computed as the percentage task completion by a participant.
- *Efficiency*, measured as: *(i)* *Speed:* time (measured in seconds) a participant takes to accomplish the task. The time taken to display-record activity is not considered; *(ii)* *Interactiveness:* number of clicks (or question to the chatbot) made by a participant to complete the task.
- *Satisfaction*, represented by the average of the answers of each participant to the Post-Task question PT.

To measure user experience, we adopted a dependent variable for each UEQ category. We compute the average of the questions belonging to a given scale for each participant [35].

#### Procedure

The evaluation procedure has been conducted in the one-to-one modality in a lab setting under controlled conditions. A supervisor recorded the sessions without intervening. Participants were first informed about the aim of the study. Before starting the experiments, participants filled in the questionnaire in Table 12, to collect their perceptions on their capacities and beliefs. Participants individually received a short tutorial on the Thea functionalities. Then we let them explore the prototype for four minutes. Participants were then given tasks, detailing the ones in Table 6 to complete. The successful completion of each task was assessed by the supervisor, who observed how the participants completed the assigned tasks, took the time, and the number of interactions. After each task, the post-task question PT was answered. Upon completion of the tasks, participants were asked to fill in again the questionnaire in Table 6, the UEQ questionnaire and an open comment question.

#### Analysis procedure

The considered questionnaire has been extracted from a validated one. We analyzed whether the questionnaire validity has been preserved as described in the Appendix. We adopted the paired two-sided Mann-Whitney U test to find out whether there is a significant difference between capacities and beliefs perceptions with the questionnaire in Table 12, before and after the experiment. In case of statistically significant difference, we apply the Cliffs distance [26] for determining the dimension of the difference. We also used boxplots to illustrate the score distributions for each response variable and report the statistical data. A similar procedure is adopted for the Usability and User Experience variables for which we compare the statistical data of SU and NSU to verify if there is a difference in their perceptions.

### Results

In the following we report the results related to the three research questions related to Empowerment, Usability and User Experience.

#### RQ1: empowerment

**Fig. 9:**
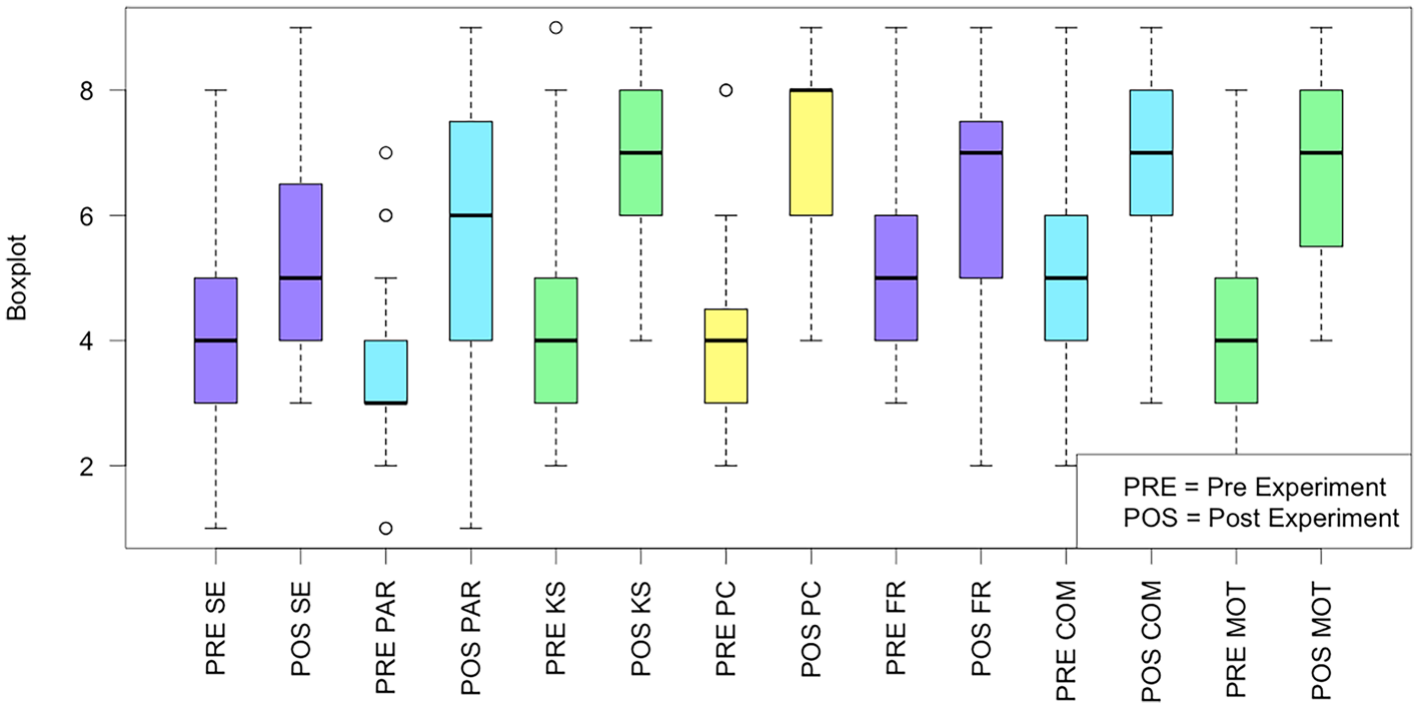
Boxplots of the Capacity-Belief Questionnaire

As shown in Fig. 9, the empowerment beliefs perceived by the participants appear to increase after the experiment. Deeper information is reported in Table 14, where the summary statistics of the Empowerment variables distribution are shown. Table 15 reports the statistical significance and the Cliff Effect size of each variable. In all the cases there is a significant statistical difference with a medium or large effect size.

**Table 14:** Statistics of the Capacity-Belief questionnaire

**Technology**	**Variable**	**Min**	**Max**	**Median**	**Mean**	**St.Dev**
PRE	SE	1	8	4	4.16	1.57
	PAR	1.0	7	3	3.48	1.36
	KS	2	9	4	4.58	1.73
	PC	2	8	4	43	1.45
	FR	3	9	5	5.10	1.56
	COM	2	9	5	5.23	1.75
	MOT	1	8	4	4.065	1.65
POST	SE	3	9	5	5.58	1.76
	PAR	1	9	6	5.90	2.04
	KS	4	9	7	7	1.65
	PC	4	9	8	7.01	1.50
	FR	2	9	7	6.36	1.59
	COM	3	9	7	6.581	1.523
	MOT	4	9	7	6.64	1.60

**Table 15:** Summary of the results of the Capacity-Belief Questionnaire

**Variable**	**p-value**	**Effect size**
Self-Efficacy	0.003	-0.426 (medium)
Partnership	5.687968e-06	-0.663 (large)
Knowledge & Skills	4.422434e-06	-0.671 (large)
Personal Control	1.964025e-08	-0.819 (large)
Feeling Respected	0.002	-0.455 (medium)
Communication	0.002	-0.445 (medium)
Motivation	7.604048e-07	-0.724 (large)

#### RQ2: usability

*Effectiveness.* All the participants successfully completed all the tasks.

**Fig. 10:**
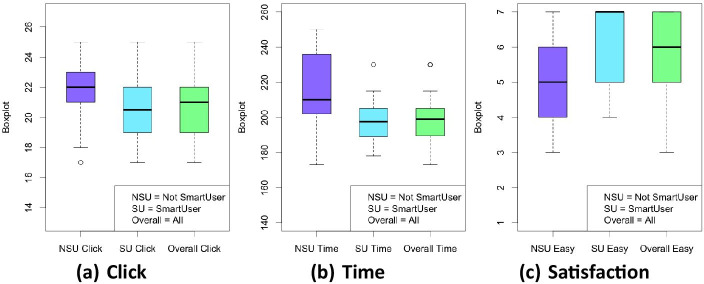
Boxplot of Click (**a**), Time (**b**) and Satisfaction (**c**) variables

*Interactiveness.* The distributions of the number of interactions appear similar in the case of SU and NSU, as shown in Fig. 10a. This is also confirmed by the statistical data in Table 16, where the summary statistics of each usability variable are reported for NSU and SU. The overall data of the sample is also resumed. Table 17 shows that the difference is not significative (p-value=0.638).

*Speed.* The time to accomplish the task appear to be larger in the case of NSU users than SU ones, as shown in Fig. 10b. This is also confirmed by the statistics data in Table 17 and by the results of the statistical significance Mann-Whitney U test (p-value=0.025) with a large effect size.

*Satisfaction.* As shown in Table 16 and by the boxplot in Fig. 10c SU appears more satisfied than NSU. This is confirmed by the significant statistical difference highlighted in Table 17 (p-value=0.029) with medium effect size (-0.444).

**Table 16:** Statistics of the Usability variables

**Technology**	**Variable**	**Min**	**Max**	**Median**	**Mean**	**St.Dev**
NSU	click	17	25	22	21.39	2.29
	Time	173	250	210	212.308	21.616
	Satisfaction	3	7	5	5.15	1.28
SU	click	17	25	20.50	20.50	2.41
	Time	178	230	197	197.44	13.544
	Satisfaction	4	7	7	6.17	1.04
Overall	click	17	25	21	20.87	2.36
	Time	173	250	199	199	13.67
	Satisfaction	3	7	6	5.74	1.24

**Table 17:** Summary of the results of the Usability variables

**Variable**	**p-value**	**Effect size**
Click	0.638	-
Time	0.025	0.483 (large)
Satisfaction	0.029	-0.444 (medium)

#### RQ3: user experience

**Fig. 11:**
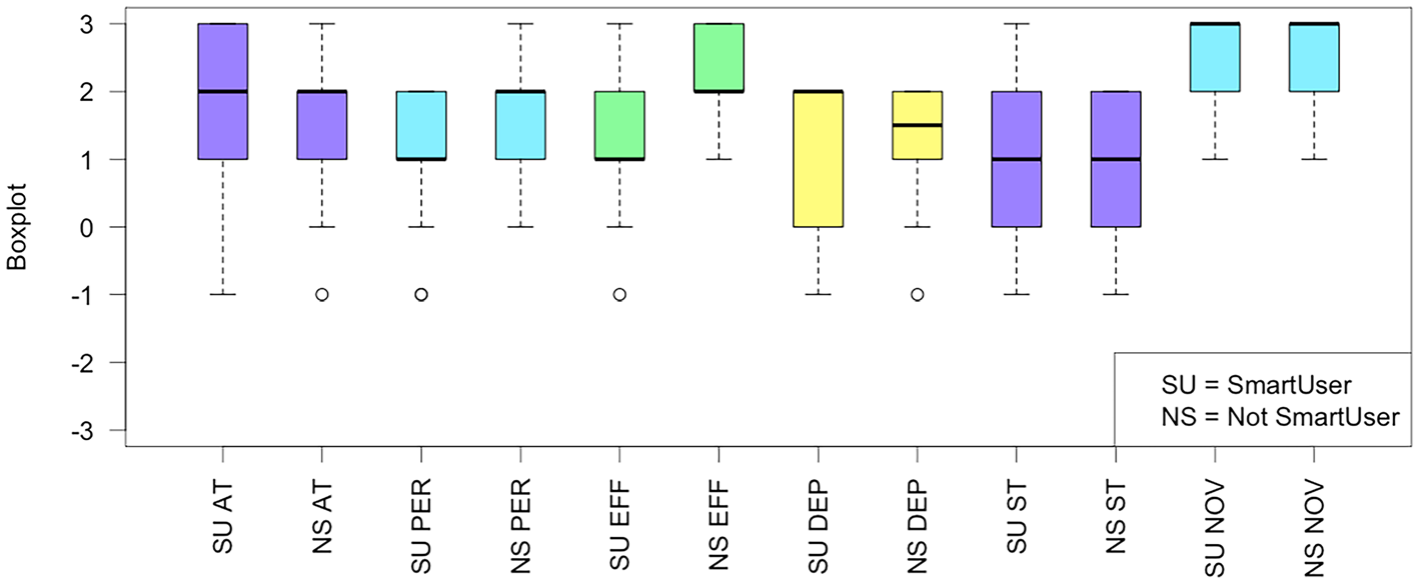
Boxplots of the UEQ Questionnaire

Concerning the User Experience, the perception is generally positive, as shown in the boxplot in Fig. 11. Statistical analysis revealed only a significant difference in the perception of Efficiency (p-value=0.036), with medium effect size (Cliff delta=-0.432).

#### Threats to validity

We report the treats to validity may affect the experiment results.

*Internal validity.* Low user experience. All the participants were new to the tool. It may be they did not understand the procedure. But generally, the Post Task questionnaire got a good result. In addition, we let participants practice with the tool before starting the experiment. The voluntary participation in the study may be a threat (motivation). All the participants were final end-users (caregivers) of a child with ASD and then motivated, as the target users should be. To limit the threat of diffusion or treatment imitations, the supervisor retained the material to avoid exchange of information by the users. Also a small sample size may be a threat to validity.

*Construct validity.* We also tried to mitigate evaluation apprehension threat by assuring participants that their information will be anonymously managed and diffused in aggregated form. A threat may be due to the absence of a baseline tool. To mitigate the restricted generalizability across constructs we considered the impact on the user smartphone use. The approach may impact other constructs we did not observe.

*Conclusion validity.* We choose tests that do not require normally distributed data, such as Mann-Whitney U test and Cliffs Delta effect size. The treatment was maintained similarly for the different participants: with the same setting and light.

*External validity.* To mitigate the external validity we considered both users with high and low familiarity with the use of smartphones. All the participants were target users of the application. Moreover, they were not informed on the aim of the study or on the research hypotheses.

## Discussion

We discuss in the following the implications of our study and conclude this section by summarizing guidelines that may be useful for developing an empowering application for the TA of a different type of patients.

### Implications and future extensions

We delineate a number of practical implications from the perspective of the different stakeholders and of the researchers to continue this work.

In this paper, we have described our work for empowering the therapeutic alliance members of a person with ASD. The aim is not a substitution of the physical relationships between the TA members, but their enhancement through a multimedia-based social sharing of methods, good practices, signs of progress, and recording of challenging behaviors. Previous researches concerned the sub-ministration of ABA therapy via tele-health, but our approach enables the increasing of empowerment beliefs of the patient’s circle, by enhancing their communication, autonomy, awareness, and knowledge.

Overall, the results of the third iteration of the prototyping phase suggest that there was a positive attitude towards the application. In particular, the empowerment perceptions of the caregivers increased for all the variables after the tool has been used. This is mainly due to the absence of a similar application. Indeed, as an example, an open comment was: *“When will this application be available?”*, or *“Where can I download the application?”*. Open comments were quite enthusiastic, and confirmed our perception of the need of this kind of application.

The technological skills of the TA members may be a possible bias. Indeed, according to EU General Data Protection Regulation (GDPR)^3^ Thea should respect the right of *Justice.*, e.g., the equal right of accessing to mHealth. To pursue this objective the application should be easy-to-use, robust, interoperable with the other healthcare systems, and accessible by all the citizens. Following this direction, in the user study in Sect. 6.4 we examined whether the application was easy to use for both smart skilled users (SU) and occasional smart users (NSU). We also add the interaction with a chat-bot for guiding the user in the selection of the app functionalities. Preliminary results were encouraging: also occasional smart users completed all the tasks, they do not have particular differences with the interaction with the application, but took more time. This is also revealed by the UX data related to efficiency: the system is perceived as more efficient by SU, even if the average values are both positive (1.782 for SU vs 1.0 for NSU). Similar consideration holds for the easy-to-use question after each task (6.17 was the average for SU, and 5.15 for NSU). Thus, the user ability in using smartphones impacts task performances. The supervisor revealed that a user had a problem using the chatbot because of her pronunciation. A training of Thea on the user pronunciations has to be inserted. UX results were positive, especially for the novelty perception, but the interaction with Thea should be further simplified for increasing efficiency.

Thea may help to solicit therapists in recording their sessions by videos, a much-felt need by the relatives and the teachers. Indeed, the therapist may take videos in a easier way, in hand-free modality. This may be enforced by recommending to add rewards both for health providers and patients/caregivers in terms of points and showing them on a community board. The adding of reputation features may motivate the health providers in sharing their competencies through video content.

It is also worth mentioning that teachers and caregivers may be stimulated by the social aspects to produce examples of their work and the signs of progress of the child, with a virtuous mechanism of emulation. Concerning the clinician, she may better supervise the work of therapists, the family involvement, and the child progress, having all this information in a unique place in a classified and organized way. All the stakeholders benefit from the possibility of recording activities and challenging behaviors in a hand-free modality. It is very difficult to take a video in a challenging situation. On the other side, all the TA members are solicited to contribute to filling in the electronic Diary of all the activities, organized by tasks.

Because the therapy for people with ASD requires to be applied for several hours a day, also patients and their circle living in rural and remote areas far from specialists or in the pandemic situation in which the distance is counseled may benefit from this kind of distance counseling. In particular, during the Covid-19 pandemic context children with ASD have to stay at home. They should continue to receive an education. Thea provides valid support to all the stakeholders for this situation.

The use of Thea has the secondary (but very relevant) effect to reduce challenging behaviors by teaching caregivers and teachers to prevent and manage them. This improves the quality of life of all the family, with a positive perception of empowerment and the reduction of the feeling of abandonment of everybody, including teachers. The multimedia contents collected by THEA also provide a trace of the progress and contribute to motivating the circle.

In Italy people with autism fall under the care of the State. School is free for all the children. One-to-one educational support is provided to children with ASD at school, depending on the ASD severity level. ABA interventions are not offered by the Italian health system which provides few hours per weeks of psychomotor therapy, or logotherapy [9]. In Italy, the practice of online counseling in the public field is absent [7]. Nevertheless, there is an increasing demand of ABA services, which are mainly privately funded by the families and performed at home after school. The communication approach supported by THEA creates a network among the circle people, involving also the teacher and the patient when enabled by her autonomy level. Thus the therapy may be applied everywhere (school or home), with a sense of community. Our system enables the structuring of the activity to perform with the possibility of organizing activities for objectives, such as learning to be autonomous in primary needs (e.g., breakfast, washing, dressing). Thus, caregivers and teachers may easier involve the child in structured activities different from her preferred solitary activities.

Another advantage of Thea is that when a teacher, a clinician or a therapist changes, her substitute may in a short time be informed on the child therapy and his challenging problems.

Concerning the end-user interaction, our study adopted a chatbot as interface. Both researchers and practitioners may be interested in investigating whether the use of other interaction modalities, such as the gestures, may improve the empowering perceptions of the target users. Also the use of wearable technologies [13] may be integrated in Thea for detecting or predicting challenging behaviours.

Patient empowerment requires the patient to be at the center of the process. In the case of children with autism, this is not always possible because the autism spectrum has several gravity levels. The circles we examined in this case study were related to patients who did not have such an autonomy level and were not able to perform activities without support. For this reason, the application developed and described in this paper seems more oriented to child relatives and caregivers. We have to extend it by examining the usability requirements also for more autonomous children.

Because the current experiment has been conducted with parents of children with ASD further studies involving the use of the system by a large sample of all the stakeholders are needed to further improve the easiness-to-use of the system.

The diffusion of a new technology/method is easier when user studies empirically show that it solves real issues [33]. The results of our study suggest that the functionalities offered by Thea may be very useful for the community of people with ASD. This outcome could increase the diffusion of such a kind of empowering approaches and the definition of new ones for other target of patients. These points are clearly relevant for both the practitioner and the researcher.

### Guidelines

This study may provide useful guidelines to identify inefficiencies and factors to be improved in the case there is the need of developing an application for enhancing the TA for a different kind of pathology to be inserted in a user-centered design process. In the following we summarize some guidelines that can be drawn by this study.

- *Guideline 1: identify the needs for actuating the behavior changes. * This may be performed by understanding the empowerment levels of the Therapeutic Alliance members and of the provider(s) of the health service. Based on the empowerment goals summarized in Sect. 5 the tools developed in this paper may be applied: the template document shown in Table 2 and Table 3 may be adopted to customize the TAT for conducting a discussion with representative users, i.e., caregivers and clinicians, respectively.
- *Guideline 2: identify the empowerment objectives and the multimedia interaction needs by using task analysis.* Analyze for each individuated task the behaviors and the capability to empower and whether the multimedia technology may support the task execution, as shown in Table 6.
- *Guideline 3: design the empowerment process by using a scenario-based description supported by a model of the process represented by an activity diagram with object-flows and swimlanes.* The $<<Multimedia>>$ stereotype may be adopted for signaling the activities requiring a more specific multimedia interaction design.
- *Guideline 4: assess the user perception of empowerment. * The assessment of the Empowerment perception before and after using the application may be useful to understand whether the application increases the capacities and the beliefs of the TA members in the management of the patient’s condition. The questionnaire adopted in this paper (shown in Table 12 and validated in Appendix A) may be adopted.

## Conclusion

In this paper, we presented an approach for supporting the empowerment of the Therapeutic Alliance of children with ASD through the definition of communication process among the alliance members with the adoption of multimedia interaction technologies. We identified the aspects that should be empowered in terms of capacities and behaviors by exploiting TAT. When evaluating the application we considered two kinds of smartphone users: expert and occasional. The results collected in the case study are preliminary, but encouraging, also for occasional users.

Multimedia applications for the empowerment of TA may be a solution for rehabilitation problems where there is a shortage of therapists and clinicians. This is particularly true in our specific contingent moment of the Coronavirus pandemic. Indeed, children with ASD stay at home, and their family members need to keep on providing the needed care to them in order not to lose the acquired abilities.

Future work may be devoted to collect all the content produced by the multimedia applications and the success stories to create a knowledge base useful for the patient community. Recommendation systems could be designed to suggest training activities to patients, under suitable safety assurance and medical control.

## Figures and Tables

**Fig. 12:**
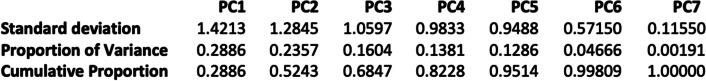
Importance of Components

**Fig. 13:**
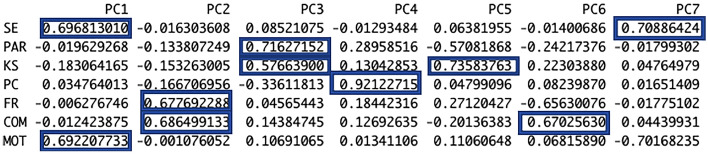
Rotation $$(n \times k) = (7 \times 7)$$

## Appendix A

Before using the data collected by the questionnaire reported in Table 12 to assess the Empowerment perception in our study, we studied its reliability. We derived it from established questionnaire [22]. Further verification was needed to understand in which measure the applied modifications preserve the reliability of the initial questionnaire. To this aim, we applied the Principal Components Analysis (PCA) on the data collected before starting the experiment for examining the factor structure of the questionnaire. Then we verified the internal reliability of the questionnaire by using Cronbach’s $\alpha$. The usual threshold for acceptability for Cronbach’s $\alpha$ is 0.7 [31].

As shown in Fig. 12, the components PC1-PC5 reached a cumulative proportion of 95% of variance. Thus, PC6 and PC7 could be excluded. We may select the variable reaching variance greater equal than 0.5, i.e., SE and MOT for PC1, FR and COM for PC2, KS and PAR for PC3, and PC for PC4. Cronbach’s $\alpha$ was 0.99 at 95% of confidence level for the selected questions in PC1; for FR and COM it was 0.58. So we assigned COM to PC6 and FR to PC2. A similar result is reached for PC3 ($\text {Cronbach's }\alpha =0.25$). We assigned KS to PC5. As we observed that SE also reach 0.70 in PC7, we decide that the questions we extracted from [22] may be used without grouping them in factors (see Fig. 13).

## Footnotes

1 https://www.autismspeaks.org/applied-behavior-analysis-aba-0

2 https://www.ueq-online.org

3 https://europa.eu/youreurope/business/dealing-with-customers/data-protection/data-protection-gdpr/index_en.htm
